# Expanding our concept of simulation in radiology: a “Radiology Requesting” session for undergraduate medical students

**DOI:** 10.1259/bjro.20220012

**Published:** 2022-10-11

**Authors:** James Hartley, Bobby Agrawal, Karamveer Narang, Edel Kelliher, Elizabeth Lunn, Roshni Bhudia

**Affiliations:** 1 Royal Papworth Hospital, Cambridge, United Kingdom; 2 University of Cambridge, Cambridge, United Kingdom; 3 Imperial College, London, United Kingdom

## Abstract

**Objectives:**

Whilst radiology is central to the modern practice of medicine, graduating doctors often feel unprepared for radiology in practice. Traditional radiological education focuses on image interpretation. Key areas which are undertaught include communication skills relating to the radiology department. We sought to design teaching to fill this important gap.

**Methods:**

We developed a small group session using *in situ* simulation to enable final and penultimate year medical students to develop radiology-related communication and reasoning skills. Students were given realistic cases, and then challenged to gather further information and decide on appropriate radiology before having the opportunity to call a consultant radiologist on a hospital phone and simulate requesting the appropriate imaging with high fidelity. We evaluated the impact of the teaching through before-and-after Likert scales asking students about their confidence with various aspects of requesting imaging, and qualitatively through open-ended short answer questionnaires.

**Results:**

The session was delivered to 99 students over 24 sessions. Self-reported confidence in discussing imaging increased from an average of 1.7/5 to 3.4/5 as a result of the teaching (*p* < 0.001) and students perceived that they had developed key skills in identifying and communicating relevant information.

**Conclusions:**

The success of this innovative session suggests that it could form a key part of future undergraduate radiology education, and that the method could be applied in other areas to broaden the application of simulation.

**Advances in knowledge:**

This study highlights a gap in undergraduate medical education. It describes and demonstrates the effectiveness of an intervention to fill this gap.

## Introduction

### The issue

Radiology is a keystone of modern medical practice,^1,2^ but many newly qualified doctors and radiologists often feel that radiology teaching at medical school is inadequate.^3–5^ Trainees and radiologists can feel that new doctors start their careers insufficiently prepared for key clinical aspects of radiology – evaluating the appropriateness of imaging, understanding how radiology departments prioritise investigations, understanding adverse risks and reactions, and communication with the radiology department.^3,6^ This lack of communication skills can result in frustration both for the radiologist and non-radiology trainee, and may result in a higher likelihood of inappropriate imaging being obtained to the detriment of patients and healthcare services, with patients receiving higher radiation doses due to unnecessary procedures.

## Simulation

Simulation training is increasingly used in innovative ways throughout medical education.^7,8^ From a theoretical standpoint, simulation can be thought of as relating to a social-constructivist and sociocultural theory,^9–12^ where learners engage in “legitimate peripheral participation” in the practice of medicine, briefed and debriefed by more knowledgeable others who help them to relate their experiences to the wider body of practice and knowledge. Within radiology, simulation is being successfully used in training postgraduates in procedural skills, with some limited but increasing use in interpretive and communicative skills.^13^ However, there is little description of training for undergraduates in the communication and decision-making skills needed to optimise the use of radiology clinically.

## Our intervention

We developed an innovative small-group simulation-based teaching session with the objective of helping undergraduate medical students gain confidence and skill in communication with radiologists, in understanding the role of the radiology department, and in evaluating the costs and benefits of imaging. We used real hospital phones and involved a senior radiologist in the delivery of the sessions in order to heighten the fidelity of the simulation and further lean into the theories of learning central to simulation training. We evaluated the impact of this approach with the goal of ascertaining whether it should be used more widely or extended into other specialities.

## Methods

### Setting and participants

Study participants were final or penultimate year medical undergraduates studying medicine at the University of Cambridge whilst on placement at Royal Papworth Hospital.

### Intervention

The radiology simulation consisted of a single 90-min session facilitated by one Medical Education fellow and one radiology consultant.

The session is designed for five students and consisted of three parts:

1. Part 1: brief/discussion of the skill (15 min)

Before the session, students are sent a session outline.

At the start of the session, the facilitator asks the students in what situations a Foundation Year 1 (a doctor in their first year after medical school, or “FY1”) would call a radiologist via telephone, accepting suggestions from the group. There is then a facilitated discussion covering key information a junior doctor would need to know when speaking to a radiologist in various situations. Minimal time is spent adding information to students’ knowledge at this stage.

2.Discussion of cases and simulation (60–70 min)

Students are given hand-outs briefly describing medical cases (Supplementary Material 1). Cases were chosen over repeated discussions with two senior radiologists and four junior doctors. The selection of cases was developed with input from consultant radiologists to represent common scenarios seen by medical trainees on-call, and to cover a broad range of specialities and basic imaging types. Each gives basic but incomplete information regarding a clinical situation in which the junior doctor would be expected to call a radiologist to arrange an investigation or intervention. It may include prompts to help the student think through the case and raise specific learning points. A summary of the six cases is shown in Table 1.

Supplementary Material 1.

**Table 1.** Summary of the six cases used in the session and key learning points

Case	Description	Learning points
Fall and CT head	An elderly patient falls on the ward at night, bumps their head, and has a GCS of around 13 when seen. It transpires they are on Warfarin.	Asking about anticoagulants; weighing up risks and benefits of radiology out of hours with radiologist; the practical use of guidelines in decision-making and communication.
Pulmonary Embolus	A post-operative patient with a complex social history is suddenly breathless and unwell.	Choosing the important information to convey to a radiologist when a lot of information is available. Conveying priority level of scan.
Urinary colic/pyelonephritis	A patient with urinary colic type symptoms also has raised white cells, CRP and raised creatinine when the students ask for bloods.	Conveying priority. Choosing the correct investigation.
Drainage of pleural effusion	A female with worsening chronic breathlessness and a large effusion on CXR needs an ultrasound-guided chest drain.	The different pieces of key information, including information about patient fitness and capacity, which need to be discussed when requesting a procedure.
Postoperative anastomotic leak	A patient becomes peritonitic and unwell after a recent bowel resection.	Conveying urgency. Choosing the right information to present when justifying a surgical scan.
A young female with pelvic pain.	A 20-year-old female attends A&E with severe pelvic pain. Both surgery and gynaecology want her to have imaging before accepting.	Discussing the correct choice of scan with the radiologist when the diagnosis is not clear. Developing awareness of practical aspects *e.g*. needing to have a full bladder for pelvic US.

The students are given five minutes to read through their case. Following this, each student is invited to act as an FY1. They are given a chance to “gather more information” – *e.g*. what could be gathered by speaking to their registrar, checking results on the computer, etc. This information is provided by the facilitator on request. They are also given the opportunity to gain input from their student colleagues.

The student is then asked to call a radiologist to request relevant radiology input. An actual hospital phone was used for this where possible. On the phone, the student talks to a radiologist. The radiologist treats the student and addresses the request as they would that of an actual FY1 in day-to-day practice. Once the discussion has reached a conclusion, the radiologist may “break character” to give the student instant feedback. This is followed by a short debrief with the group, with a focus on communication skills, and with reference to appropriate guidelines such as the American College of Radiology’s Appropriateness Criteria^14^ or the National Institute for Health and Care Excellence guidelines.^15^

3.Summary and closure (5 min)

After all students have had an opportunity to simulate a request, there is a brief closing section where key learning points are re-iterated and any information missed from the opening brief is highlighted. Students are directed to any key resources which have not already come up for further reading. Questions are asked and answered.

A summary of key learning points is sent to the students after the session.

**Figure 1.**
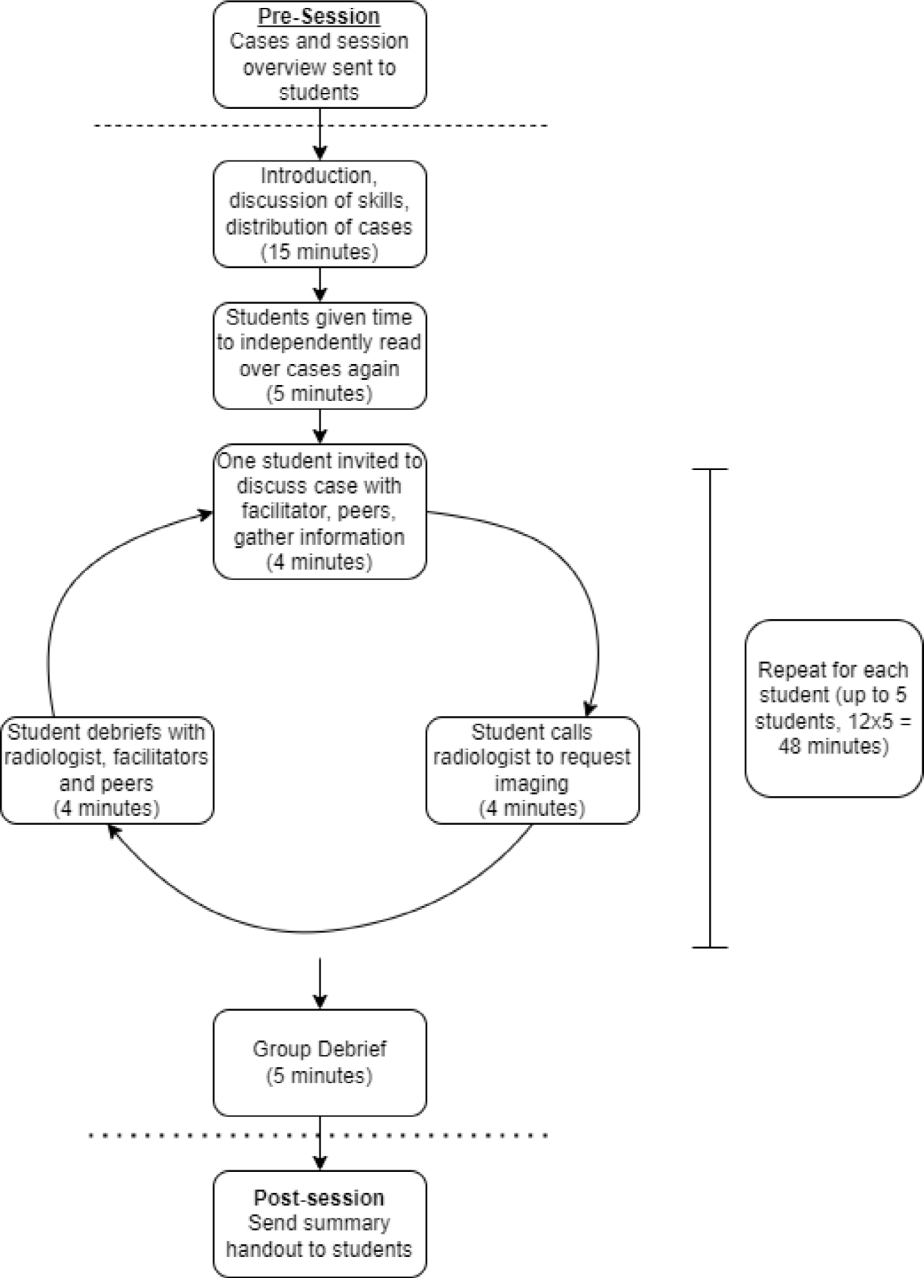
Summary of session structure

A breakdown of the session is given in Figure 1.

### Participants and group sizes

The intervention was delivered to 99 students over 24 sessions. Of these, 39 were in their penultimate year and 60 in their final year. The group size was between two and five students, with a median of 4 and mode of 5.

### Data collection

Paired data were collected before and after the session using printed questionnaires. These collected quantitative data pertaining to the student’s confidence with various aspects of requesting radiology using Likert scales, and qualitative data concerning the student’s chief concerns regarding requesting radiology and impressions of the session. We asked students eight questions relating to their perceptions of and confidence in requesting imaging. These were:

- How important do you think it is to be proficient at discussing scan requests with a radiologist?
- How would you rate your knowledge about discussing scan requests?
- How would you rate your comfort in communicating scan requests over the telephone with a radiologist?
- How would you rate your confidence in discussing scan requests with a radiologist?
- How would you rate your ability to pick out key information from a patient’s notes?
- How would you rate your baseline ability to determine what additional information you may need about a patient prior to discussing a scan?
- How would you rate your baseline ability to synthesise a scan request in a logical manner?
- How would you rate your baseline ability to deal with a scan request being declined by a radiologist?

Note the term “scan” is often used generically to refer to imaging in the UK context. Likert scales from 1 (Not important at all etc) to 5 (Extremely important etc) were employed. These questions were repeated before and after the session to assess for improvement. The questionnaire as printed is available as Supplementary Material 2.

Supplementary Material 2.

### Data analysis

Quantitative data were analysed using Microsoft Excel and the XRealStats Excel add-on.^16^ A paired Sign test was used to investigate the hypothesis that attending the teaching session changed the median Likert scale scores for each question. A Mann-Whitney U-test was used to investigate for the differences between the final year and penultimate year students’ responses.

Qualitative data were analysed using a basic Thematic Analysis approach.^17^ Qualitative data were examined independently by two researchers (JH and EL), who independently produced “codes” to label recurring ideas within the data. The two researchers recursively discussed their codes and returned to the data until an agreed set of codes was reached. These were then reviewed and arranged by common elements into identifiable “themes”, broad concepts which together appear to give meaning to the qualitative data as a whole.

### Ethical approval

This study was approved by the Royal Papworth Hospital R&D unit as not requiring further ethical review.

## Results

### Quantitative data

### Prior experience with requesting radiological investigations

**Figure 2.**
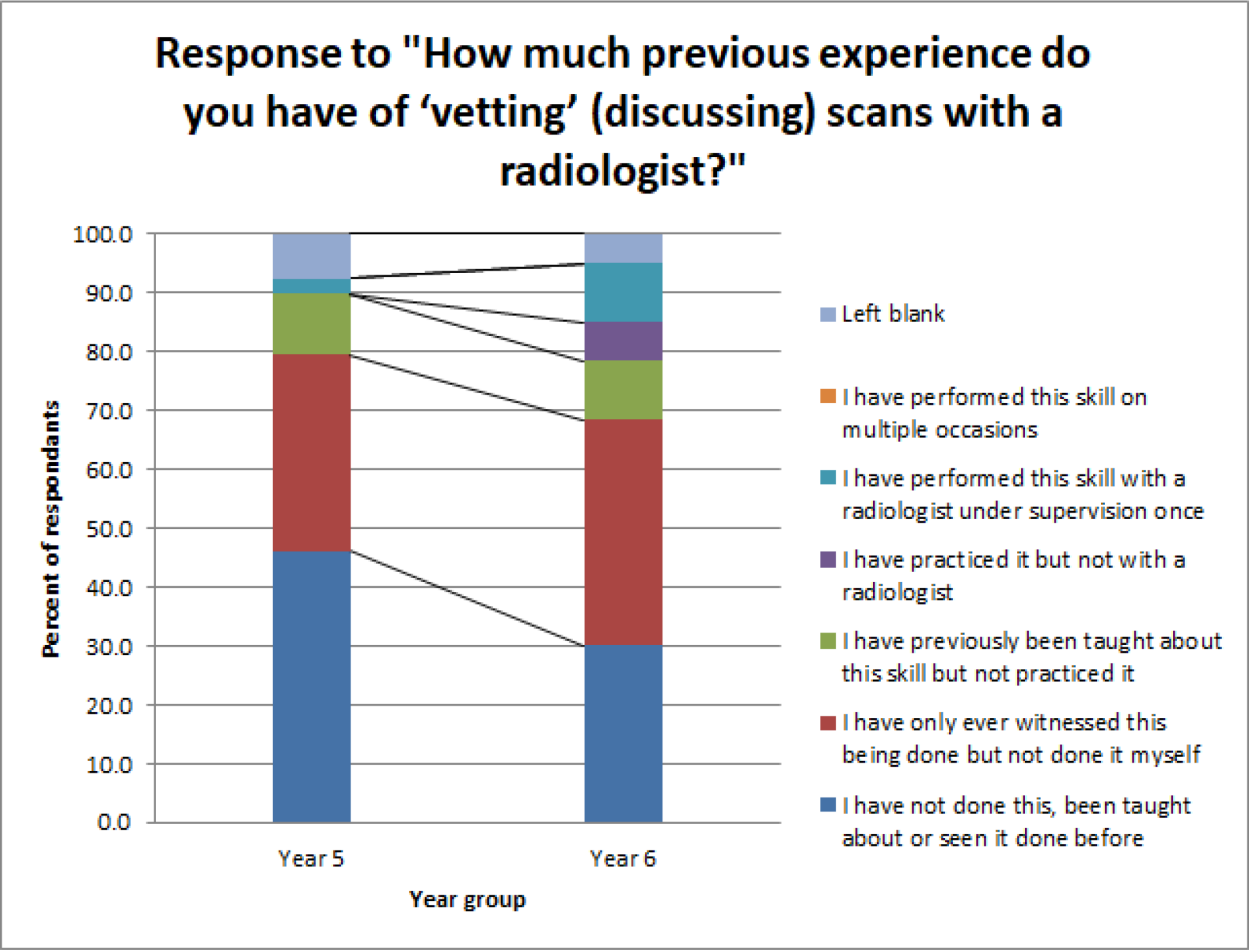
Student responses regarding prior experience with teaching about requesting radiology.

We asked students to evaluate their prior experience with booking radiological investigations, choosing from a range of options. 36.7% of students reported that they had never seen imaging being requested, and a further 34.4% reported that they had witnessed imaging being requested but never been taught about it or practised it. Amongst year six students only, these numbers became 30.0 and 38.3% (Table 2 / Figure 2).

**Table 2.** Student responses regarding prior experience with teaching about requesting radiology

Response to “How much previous experience do you have of ‘vetting’ (discussing) scans with a radiologist?”	Year 5 (No.)	Year 5 %	Year 6 (No)	Year 6 (%)
*I have not done this, been taught about or seen it done before*	18	46.2	18	30.0
*I have only ever witnessed this being done but not done it myself*	13	33.3	23	38.3
*I have previously been taught about this skill but not practised it*	4	10.3	6	10.0
*I have practised it but not with a radiologist*	0	0.0	4	6.7
*I have performed this skill with a radiologist under supervision once*	1	2.6	6	10.0
*I have performed this skill on multiple occasions*	0	0.0	3	0.0
*Left blank*	3	7.7	3	5.0
*Total*	18	46.2	18	30.0

### Changes in confidence over the session

The results of the eight Likert scale questions are shown in Table 3. Pre-intervention students rated themselves as unconfident (less than three out of five) 72.9% of the time averaged across all questions. Questions which focused on communication skills received somewhat lower ratings. The proportion of students rating themselves as two or less was 83.8%, 80.8% and 88.9%, respectively, for “comfort communicating scan requests over the phone”, “confidence discussing scan requests with radiologists” and “dealing with scan requests being declined”, respectively, compared to 42.4%, 66.7%, 71.7% and 75.8% for “ability to pick out key information from patient’s notes”, “ability to determine further relevant information”, “baseline knowledge” and “ability to synthesise a request in a logical manner”, respectively.

**Table 3.** Results of Likert scales from questionnaire. After applying a Bonferroni correction significance is achieved at *p* < 0.003.

Final year student results					
Question	Pre-teaching mean	Pre-teaching median	Post-teaching mean	Post-teaching median	Paired Sign Test *p*-value
Importance of presenting	4.70	5	4.88	5	0.00342
Baseline knowledge	2.28	2	3.95	4	>0.0001
Comfort over the phone	1.93	2	3.97	4	>0.0001
Confidence discussing	1.93	2	3.78	4	>0.0001
Ability to pick out info	2.59	3	3.95	4	>0.0001
Determine further info needed	2.14	2	3.88	4	>0.0001
Synthesise logically	1.98	2	3.85	4	>0.0001
Deal with rejection	1.70	2	3.05	3	>0.0001
Penultimate year student results					
Question	Pre-teaching mean	Pre-teaching median	Post-teaching mean	Post-teaching median	Paired Sign Test *p*-value
Importance of presenting	4.74	5	4.89	5	0.227
Baseline knowledge	1.95	2	3.89	4	>0.0001
Comfort over the phone	1.54	1	3.95	4	>0.0001
Confidence discussing	1.59	1	3.89	4	>0.0001
Ability to pick out info	2.72	3	3.97	4	>0.0001
Determine further info needed	2.26	2	3.87	4	>0.0001
Synthesise logically	1.84	2	4.05	4	>0.0001
Deal with rejection	1.64	1	3.22	3	>0.0001

There was a significant increase in the median score for every question asked for both year groups with the exception of the “Importance of presenting” question, with a significant value of *p* < 0.003 after applying a Bonferroni correction to account for the multiple comparisons. The largest effect was seen in how students rated their “Comfort in communicating scan requests over the phone”, with a mean increase of 2.03 points on the five-point Likert scale for the final year students and 2.41 points for the penultimate year students. The smallest effect sizes were seen in students’ ratings of their “Ability to pick out key information from a patient’s notes” and their “ability to deal with a scan request being declined” – both expected, as neither of these elements were key goals of the teaching session. In the former case, students also started with a higher level of confidence than in any other question, meaning the capacity for improvement was smaller. Post-intervention, students rated themselves as unconfident (less than three out of five) 3.7% of the time when averaged over all questions. Within this, 26.3% of students rating themselves as unconfident in “dealing with rejection”, 2% in “synthesising information”, and 1% in “picking out information from notes”. No students rated themselves as unconfident in their level of knowledge regarding imaging requests, their confidence over the phone, their confidence in discussing with radiologists or in their ability to determine important information post intervention.

After accounting for the multiple comparisons (and thus a significance value of *p* < 0.003), a Mann-Whitney U-test revealed no difference between the scores from final year and penultimate year students for any question either pre-intervention or post-intervention. Differences with *p* values less than 0.05 were students rating of their pre-intervention level of baseline knowledge (an average score of 2.28 out of 5 in final-year students and 1.95 out of 5 in penultimate year, *p* = 0.0167), their preintervention level of comfort over the phone (1.93 *vs* 1.54, *p* = 0.00487) and their confidence in discussing imaging requests (1.93 *vs* 1.59, *p* = 0.0254). Post-intervention all *p* values were greater than 0.1.

### Overall impressions of the session

Ninety-five out of 99 students (96.0%) said they fulfilled their objectives for this session. The remaining four left this question blank. Eighty-four students indicated they would “definitely recommend” this session (five on the Likert scale); a further ten responded with a four on the scale and one with a three. Four students did not respond.

### Qualitative data

### Students’ ideas and concerns pre-session

We asked students to tell us their key objectives going into the session. Four key themes emerged:

1. The desire for *personal development* expressed in terms of *confidence and comfort*
2. The desire to develop focused *communication skills*
3. The desire for *specific items of knowledge*
4. The desire for *feedback in a safe environment*

### Personal development and confidence

Forty students out of 99 mentioned wanting to gain confidence in interacting with radiology as part of their aims. This was often paired with concerns about performing the task once qualified – one student wrote that they aimed to “Become more confident in understanding and discussing radiology cases to reduce worry when performing this as an FY1”. Three students added that they wanted to develop more confidence interacting with colleagues over the phone. Seven specifically mentioned hope that the teaching would help them “handle rejection” or “handle a scan being denied”.

### Communication skills

Twenty-seven of the 99 students mentioned improving their ability to communicate as a priority. Frequently expressed goals were a desire to “*learn a structure”* for a request and to make communication *concise* and *clear*.

### Points of knowledge

Frequent goals were:

- To understand *what information* must go in a request
- To understand *when* to call a radiologist
- To understand *and overview of how scans are requested and the role of the radiology department within this*

### Feedback and safety

Nine students mentioned that a key goal for the session was to get feedback from facilitators and peers on their performance.

### Perceptions of the session after the teaching

Ninety out of 99 students wrote something in response to being asked what was good about the session. Their responses can be divided into two main themes, with several subthemes:

1. Appreciation of how the session was set up and ran a) A supportive and interactive learning experience (*e.g.,* “gave opportunity to practice a commonly needed skill in a secure environment”) b) Appreciation of realism and experiential learning (“Real life practice phoning the radiologist instead of just the theory”) c) Appreciation of the specific materials and cases chosen (“the cases were all very different and had clear learning points”).
2. Satisfaction at having achieved specific goals a) Feelings of personal and professional development – that they were better prepared to be junior doctors after the session (“Great to have the opportunity to feel like we know how to master the basics that will carry through to making us better lifelong clinicians”) b) Specific pieces of knowledge they took away (“helpful to learn about specific info you need *e.g*. can they sit up, creatinine for contrast, clotting for biopsies/drains”).

Only 54 students suggested improvements. Fourteen suggested “more of the same” – either longer sessions or further similar sessions. Fourteen remaining suggestions focused on technical aspects of specific sessions – for instance, some students were not aware of the post-session hand out and suggested there should be one, or suggesting the time be apportioned differently, or the session held at a different time of day. Other suggested improvements could be grouped into two themes:

1. The desire for increased challenge – particularly practice of rejection or a less “friendly” radiologist (“radiologist can be meaner”, “have rejected scans, have a less nice radiologist --> more like real life?”). This was the most common suggestion for improvement (nineteen of the fifty four students with suggestions).
2. Seven students expressed the desire for further increases to realism (*e.g.,* “add high fidelity cases *i.e.* Electronic Patient Record fake notes and bloods”).

Of the 45 who did not suggest improvements, 17 explicitly wrote that they thought nothing could be improved. The remainder left the section blank.

## Discussion

### Need for this teaching session

Seventy-three percent of our cohort of final and penultimate year medical students reported only ever-witnessing the skill of discussing imaging requests with radiology, and never having either been taught about it or practiced it. Amongst final year students alone, this fell to 68%. On average, 72.9% of students rated their confidence as two out of five or less for the various aspects of requesting imaging we asked about. These findings are comparable to those of Simelane and colleagues.^6^ Working with newly graduated doctors, they found 68% of participants rated their “Preparedness for interacting with the radiology department during their intern year” as “Somewhat unprepared” (18%), “Unprepared” (37%), or “Very unprepared” (11%). Moving away from communication skills specifically, Nyhsen et al found that 64% of a similar cohort of junior doctors in the UK felt that there had been “too little exposure to radiology” at medical school.^18^ In New Zealand, Subramaniam et al. found that 58% of doctors in their first year after graduating felt they had not had adequate radiology teaching for their work.^19^

Taken together, our results and the results of these other studies suggest that a large proportion (33–68%) of final year students and newly graduated doctors feel that their training in radiology has not been adequate. Our study somewhat expands on this by defining further what aspects of interaction with radiology students feel least confident about. Our participants rated their confidence as lower when questions were more directly rated to communication and interaction with radiologists rather than knowledge or skill with manipulating information. Notably, when asked about radiology in general rather than “interaction with the radiology department” the respondents in Simelane’s study were notably more confident, with 67% of them reporting that that had received adequate radiology instruction during their undergraduate training, and 55% of them reporting “adequate” radiology knowledge. This may indicate that communication skills and interaction with radiologists is a particular area that any increase in radiology teaching for undergraduates might focus on. However, we did not ask students about their confidence in “interpreting images” to compare, so this conclusion does require further support.

### Success of the teaching session

Our teaching session created significant increases in confidence in every aspect of requesting we investigated: students rating of their confidence in the various aspects of requesting imaging changed from 2.02 to 3.93 out of five, and the proportion rating themselves as unconfident (2 out of 5 or less) changed from 72.9 to 3.7% (both results averaged across all questions asked). This was mirrored in the qualitative data, where an increase in confidence was amongst the strongest themes in student responses an outcome of the session.

The success of the session may stem from various factors. The small-group format may be important. Students consistently rate small-group case-based teaching as incredibly useful. The format provides several benefits through high levels of interaction between the facilitator and students and between the students themselves.^20^ It provides a safe environment with the appropriate social support for high level learning^21^ – something which did indeed come through in the student’s descriptions of what was successful about the session.

The chosen content of the teaching session is likely also important. Teaching in radiology often focuses on interpretation of imaging more than communication and practical elements.^22–24^ These topics are essential; however, in undergraduate medical students Murphy et al found that teaching on image interpretation and anatomy alone does little to impart understanding of how a radiology department functions, even when small-group seminars are employed.^25^ Teaching which focuses on communication skills such as this would serve to complement and complete important teaching on image interpretation and anatomy. A session with the explicit objective of teaching the clinical skill of interacting with the radiology department is to our knowledge novel.

Finally, the use of simulation is likely important. The realism of the teaching was emphatically appreciated by the students, and one of the few suggestions for improvement was to increase the fidelity of the simulation further. Our use of realistic patient cases and conversations with other clinicians over the phone to train communication between clinical colleagues appears to be novel as well as successful. This could easily be extended to other clinician to clinician conversations between junior doctors and other departments.

### Limitations

Our work is best viewed in the context of its limitations.

This work was done in the setting of a single hospital in a single UK medical school. The University of Cambridge follows an academic course structure of two pre-clinical years, an intercalated BSc year, and three clinical years. It may be that in other settings student exposure to teaching is different resulting in different concerns.

We collected data immediately pre- and post-session, with a focus on changes in student confidence. A more rigorous evaluation of teaching would also assess change in student knowledge and behaviour over a longer time period.^26^ In the future, repeating assessment of students after a delay, using before and after quizzes as well as Likert scales, or following students up into their first qualified years would be valuable.

### Future directions

Our work here suggests several future directions. It would be possible to develop further cases and run follow up sessions with the same students. Student feedback suggests that extra practice would be appreciated, and that there is still value to be gained simply by expanding these sessions.

This teaching is relatively resource intensive. A single session for five students requires 90 min of time from the primary facilitator and one hour of time from the radiologist. However, polls of radiologists suggest that 70% of radiologists teach, and amongst those who do not, 76% would like to; 62% of those who would like to but do not say that they only do not because they have not been asked.^27^ This teaching might be both a valuable use of time for those radiologists who already teach and an opportunity to involve new educators.

Finally, the same teaching format could be used to teach interprofessional communication skills in diverse other situations. This study shows the value of arranging teaching with such skills as the goal. Situations could include interteam referrals, phone calls to General Practioners (GPs) for more information on a patient, or phone calls to consulting specialities such as microbiology or haematology for advice. Thus, there is a rich set of opportunities for this successful teaching format to be used in diverse settings.
