# Supplementary material for: Expanding our concept of simulation in radiology: a “Radiology Requesting” session for undergraduate medical students
Source: BJR Open. 2022 Oct 11;4(1):20220012. doi: 10.1259/bjro.20220012 (PMC10958647; doi:10.1259/bjro.20220012)
Supplement: Supplementary file 1 — Supplementary Material 1. [file bjro.20220012.suppl-01.docx]

**Appendix 1 – Case handouts used in session**

The session used up to six cases describing fictional patients, each designed to bring forward different learning points, as described in the main manuscript. Each case has both *student* and *instructor* versions, the latter containing additional information only available to the student upon request.

Below, additional text only given on the instructor copy is given in red.

**CASE 1 – Fall and CT head**

Mr Flover (Hospital number 345098) is a 77 year old man who came into hospital with a chest infection. He has a background of benign prostatic hyperplasia, hypertension, atrial fibrillation and very mild cognitive impairment from the beginnings of dementia (no specified type currently) which manifests as forgetfulness. He usually lives in warden controlled accommodation, and washes and dresses himself, as well as doing most of his own cooking and shopping. He has been in hospital for 5 days and has been responding well to co-amoxiclav; whilst he was initially somewhat disoriented and delirious, his daughter has reported after visiting him yesterday and again this morning that he is back to his baseline.

You are bleeped to come and see him early in your night shift because he has had a fall. The nurse reports that he was trying to mobilise to the bathroom unassisted and lost his balance - the fall itself was unwitnessed as she had been looking the other way. She heard a bump and found him lying in the door of the bathroom. They did a set of observations whilst he was on the floor and then used a hoist to help him back into the bed. His initial observations showed a HR of 90, RR of 18, BP of 132/72 and temperature of 37.0^o^C. They report he seems tired.

*What will you do after getting this call?*

You go to see the patient, arriving perhaps 3 or 4 minutes after the initial call. His eyes are closed when you approach him, but open when you address him. You do notice that when you stop talking to him he tends to drift off again and his eyes become closed. He is clearly disorientated, and denies having fallen. He asks you what you are doing in his house, and tells you to go away so that he can catch an important train, before drifting off a little and closing his eyes until you talk to him again. He lifts his arms up when you ask him to, and limited testing does not reveal any neurological deficit. On inspection you find an abrasion on the left side of his forehead. His pupils are currently equal and reactive to light.

*Consider what other information you need - you will have an opportunity to ask for it at the teaching session.*

*Will you request any imaging? If so, you will need to discuss it with a radiologist since it is out of hours, especially if you want it urgently. Plan your presentation to the radiologi*st.

Other information (will be hidden from students before sending them the case, and given to them on request):

- Drug chart: Tamsulosin, WARFARIN, Co-amoxiclav, ramipril, bisoprolol
- Bloods: INR 2.9, WCC 10 (decreasing over the last few days from a high of 18), CRP 20 (decreasing from a high of 250), Hb 131, platelets 400, CR 110 (baseline for him), electrolytes all in normal ranges.

**CASE 2 – Pulmonary Embolus**

Mrs Johannes (Hospital number 562221) is a 60 year old lady here in Papworth for a coronary artery bypass graft, which she had eight days ago. This was performed because of a long history of ischaemic heart disease, mostly angina, though she also had an NSTEMI in 2015. She lives at home with her husband and two cats; they had three sons, one of whom lives locally and visits regularly. She is type 2 diabetic but on diet control. She has hypertension usually treated with ramipril. She is somewhat overweight, weighing 86kg.

Her recovery from the operation has been a little rocky. She was quickly stepped down from ITU, but has been having quite a lot of pain from the wound on breathing and discouraging her from trying to work with the physios etc to mobilise. She remains on morphine to help with the pain.

It is about 2.30pm. You have been called to see her because she has been feeling more breathless. The nurse reads you her observations over the phone – her sats are 91% on room air (they were 98% this morning), her heart rate is 103bpm, her blood pressure is 104/70 (it is usually around 110-120 systolic) and her respiratory rate is 26. You reach the patient. The normally verbose Mrs Johannes looks unwell and out of breath. She struggles to speak to you for long but you manage a short history. She says that she was feeling ok this morning – somewhat better than yesterday – but then this afternoon she felt more breathless over the course of a couple of minutes she thinks. The breathlessness started about 10 minutes ago. This is accompanied with a little chest pain on the left side of her chest, different in nature to the pain from the operation; she says it is sharper, and worse on inspiration.

*Think now about your differential diagnoses and next actions. Write down your thoughts before continuing.*

You apply high flow oxygen. You quickly think of some differentials. You get some help - perhaps from other juniors on the ward, perhaps the critical care outreach team, perhaps you put out a medical emergency call if you are sufficiently worried (very ok to do!). You ask the nurse to get the ECG machine and one of your helpers to take and run an ABG whilst you do a quick examination of the patient. You also ask someone to arrange a portable chest X ray.

Her airway is clear. Her breathing is rapid and somewhat shallow, but when you listen to the chest you can hear no added sounds. Her pulse is rapid and somewhat thready. Her cap refill is about 3 seconds. Heart sounds are normal. Her GCS is 15 and a glucose comes back as 7. She can move both her arms and her legs apparently normally. The midline sternotomy wound looks clean. There are no chest drains still in situ. Her abdomen is soft and non tender. Her left leg is somewhat swollen - certainly bigger than the right - tender, and erythematous. Her current medications are aspirin 300mg OD, clopidogrel 300mg OD, ramipril 1.25mg OD, bisoprolol 2.5mg OD, enoxaparin 40mg S/C OD and atorvastatin 40mg OD. Her most recent bloods show an Hb of 124, WCC of 9, CRP of 12 (it was raised post surgery but has fallen consistently), normal electrolytes and a creatinine of 84.

The ABG comes back. The pH is 7.46, pCO_2_ is 4, and PO_2_ is 9 (on oxygen). The lactate is 2.8. The electrolytes match that on the formal bloods. The portable CXR is also taken and you look at it on the machine’s monitor - it is shown below.


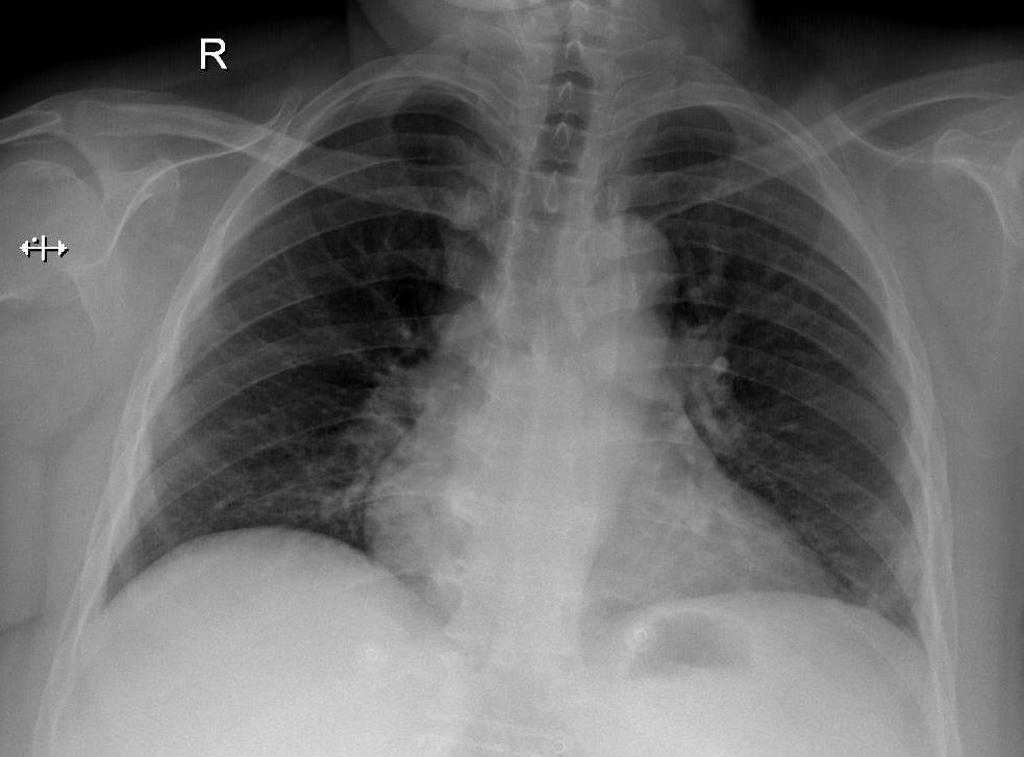


The ECG shows sinus tachycardia.

*What is your differential now? What are the next actions you take?*

*What scan will you book next? You request the scan on the hospital’s system, and now need to discuss it with a radiologist to get it urgently. Plan your presentation to the radiologist.*

**CASE 3 – Urinary colic/pyelonephritis**

You are working in A&E. A 42 year old man, Mr Lickol (Hospital Number 982201), comes in complaining of abdominal pain. The pain is waxing and waning and crampy, and localised mostly to his right flank with radiation down to the groin. He also appears unwell - sweaty and somewhat pale. He is mildly tachycardic at 94bpm, and pyrexial at 37.9^o^C. His resp rate is 18, and his blood pressure is 123/74 (he thinks that is about normal for him).

He has very little past medical history - he had asthma when he was young, which was treated with a “blue inhaler”, but that has not troubled him for years, and he takes no other regular medications. He is a little overweight, and feels that his diet could be healthier, but has recently joined a gym to try and lose some weight. He is a publican.

*What other investigations and information do you want before thinking about radiology? You will have an opportunity to ask for these before calling the radiologist. Will you prescribe any medicine etc?*

*What scan will you book? Prepare your discussion with the radiologist now.*

Additional information (hidden from medical students but available on request)

Ix:

- Urine dip: 2+ blood, 2+ leukocytes, 1+ nitrates
- Bloods: WCC 14, CRP 150, Hb 134, platelets 500, Na 138, K 4.0, Creatinine 180 (no previous on file)
- Coagulation normal
- Abdo XR normal
- CXR normal
- ABG - slight hyperventilation

**CASE 4 – Drainage of pleural effusion**

You are an FY1 on a general medical ward. You are looking after Mrs Petey (Hospital number 900232), a 63 year old woman with a background of COPD who has come in with progressive breathlessness. She normally can look after herself and go to the shops with minimal breathlessness, but over the last month has become increasingly breathless on exertion until now she gets very out of breath walking across the room. Her chest x ray is shown below.

Your registrar asks you to arrange for the radiologists to insert a chest drain under ultrasound guidance.


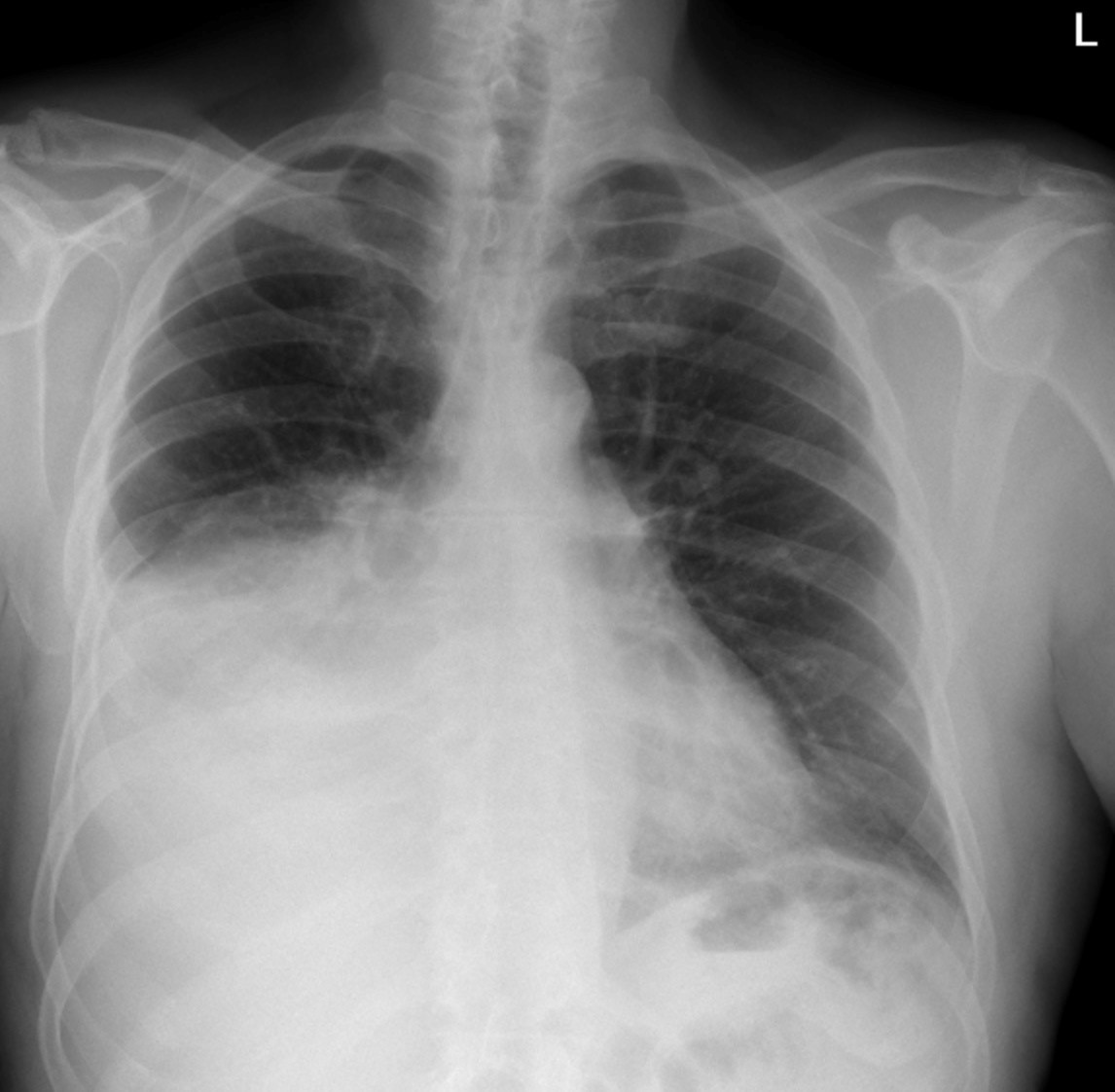
*Consider what other information you need - you will get an opportunity to ask for it before speaking to the radiologist.*

*Plan your discussion with the radiologist.*

Further information (to be hidden from student until they ask for it):

Background: Recurrent exacerbations, heavy smoker (on going), one previous NSTEMI four years ago

Medication: Aspirin 75mg OD (last taken 8am this morning), bisoprolol, atorvastatin, ramipril, salbutamol inhaler, tiotropium and salmeterol inhalers

Bloods: COAGULATION: APPT 35s, PT 13.2s. Platelets 310. WCC 11, Hb 108. Electrolytes normal. Creat 90. CRP 77. Correct calcium 2.7.

**CASE 5 – Postoperative anastamotic leak**

Mr Jones (Hospital number 673308) is a 68-year-old male who was admitted via A+E due to abdominal pain. He came in overnight after suffering from severe abdominal pain, which had been ongoing for 24 hours. He was unable to pass wind or stool and his abdomen was significantly distended when he was examined. He has a past medical history of hypertension, gout, and hyperthyroidism and takes 25mcg levothyroxine, 5mg ramipril, and allopurinol. He lives at home with his wife and is normally independently mobile. He weighs 90kg and is a non-smoker, non-drinker. He has a family history of ulcerative colitis and ovarian cancer. General surgeons came to see the patient and requested a CT abdomen pelvis, which showed a significantly sized mass in the sigmoid colon. The patient was then taken to theatre for a Hartmann’s procedure and then transferred to ITU post operatively.

Whilst on ITU, the patient recovered well and was started on TPN before progressing on to light sips of water. He was then transferred to the ward and a soft diet was commenced.

6 days after his operation, you are called to see him over concerns in regards to pain and a fever he has developed over the last few hours. The patient is reporting significant pain over his lower abdomen and nurses say he has become increasingly hot, sweaty and clammy over the last 4 hours. His temperature is noted to be 39 degrees Celsius. The patient explains that the pain is sharp in nature and that he feels his abdomen is getting bigger and bigger. He has been unable to pass any faeces or flatus for the last 4 hours.

You ask the nurse for a set of observations:

BP 107/80

HR 101

O2 saturations 95% on air

RR 20

On examination, you find that the patient is tender all over his abdomen and his abdomen is significantly distended. The patient also has absent bowel sounds. His wound from the operation looks ok with no discharge or any evidence of wound dehiscence.

*Think now about your differential diagnoses and next actions. Write down your thoughts before continuing.*

You apply high flow oxygen, give the patient analgesia, and start him on paracetamol for his temperature. You start him on fluids and you call the surgical registrar. You explain what has happened and he asks you to book a scan.

*What is your differential now? What are the next actions you take? What more information do you want*

*What scan will you book next? You request the scan on the hospital’s system, and now need to discuss it with a radiologist to get it urgently. Plan your presentation to the radiologist.*

**CASE 6**  **- A young woman with pelvic pain**

You are working in A+E. An 18-year-old female, Sonya Tan (Hospital number 775924) comes in due to significant pain in her abdomen, which started around 6 hours ago. The pain has gradually increased in severity and it is now unbearable which is why she has presented to A+E. The patient says the pain was also associated with some discharge she has noticed PV. You examine the patient and find that her abdomen is significantly tender especially over the lower part with some distension. Her bowel sounds are normal and there are no other findings on examination. Due to the history of discharge, you perform a vaginal examination (with a chaperone present), which was unremarkable.

*What is the single most important investigation you can perform at this stage? What other initial information do you want? Write down your thoughts before reading the next page.*

You perform a series of investigations:

Pregnancy test negative

WCC 15.4

CRP 176

Hb 111

U+E Normal

LFTs Normal

Her observations are as follows:

BP 119/80

HR 107

Oxygen saturations 96% on air

Temperature 37.5

*Who would you call at this stage? What team do you think this patient should be referred to? Write down an answer before reading on.*

You make the decision to call general surgery who decline the referral until gynaecology have seen the patient. The patient is then referred to gynaecology who would like you to book a scan.

*What is your differential now? What are the next actions you take?*

*What scan will you book next? You request the scan on the hospital’s system, and*

*now need to discuss it with a radiologist to get it urgently. Plan your presentation to the radiologist.*

Additional information:

- Nature of discharge: consistent with Pelvic Inflammatory Disease
- She is sexually active – one boyfriend, usually use condoms
