# Supplementary material for: Expanding our concept of simulation in radiology: a “Radiology Requesting” session for undergraduate medical students
Source: BJR Open. 2022 Oct 11;4(1):20220012. doi: 10.1259/bjro.20220012 (PMC10958647; doi:10.1259/bjro.20220012)
Supplement: Supplementary file 2 — Supplementary Material 2. [file bjro.20220012.suppl-02.docx]

**Appendix 2 – Questionnaire**

The below questionnaire was printed and given to students at the start of the session to fill out the “pre-course” section. They then filled out the “post course” section at the conclusion of the session. Results were entered into Microsoft Excel by researchers.

**Radiology Presentation Skills: Pre-course questionnaire**

Q1) How much previous experience do you have of ‘vetting’ (discussing) scans with a radiologist? (Please circle as appropriate)

I have not done this, been taught about or seen it done before

I have only ever witnessed this being done but not done it myself

I have previously been taught about this skill but not practiced it

I have practiced it but not with a radiologist

I have performed this skill with a radiologist under supervision once

I have performed this skill on multiple occasions

Q2) How important do you think it is to be proficient at discussing scan requests with a radiologist? *(1 = Not at all important, 5 = Extremely important)*

1       2     3     4     5

Q3) How would you rate your baseline knowledge about discussing scan requests?

*(1 = No knowledge, 5 = Know everything needed)*

1       2     3     4     5

Q4) How would you rate your baseline comfort in communicating scan requests over the telephone with a radiologist? *(1 = Not at all comfortable, 5 = Extremely comfortable)*

1       2     3     4     5

Q5) How would you rate your baseline confidence in discussing scan requests with a radiologist?  *(1 = Not at all confident, 5 = Extremely confident)*

1       2     3     4     5

Q6) How would you rate your baseline ability to pick out key information from a patient’s notes?

*(1 = Not at all able, 5 = Extremely able)*

1       2     **3**     4     5

Q7) How would you rate your baseline ability to determine what additional information you may need about a patient prior to discussing a scan?  *(1 = Not at all able, 5 = Extremely able)*

1       2     3     4     5

Q8) How would you rate your baseline ability to synthesise a scan request in a logical manner?

*(1 = Not at all able, 5 = Extremely able)*

1       2     3     4     5

Q9) How would you rate your baseline ability to deal with a scan request being declined by a radiologist?  *(1 = Not at all able, 5 = Extremely able)*

1       2     3     4     5

Q10) Did you manage to read your assigned case prior to the session?           Yes   No  N/A

Q11) What do you hope to gain from the session/ what are your objectives?

**Radiology Presentation Skills: Post-course questionnaire**

Q1) How important do you think it is to be proficient at discussing scan requests with a radiologist? *(1 = Not at all important, 5 = Extremely important)*

1       2     3     4     5

Q2) How would you now rate your knowledge about discussing scan requests?

*(1 = No knowledge, 5 = Know everything needed)*

1       2     3     4     5

Q3) How would you now rate your comfort in communicating scan requests over the telephone with a radiologist? *(1 = Not at all comfortable, 5 = Extremely comfortable)*

1       2     3     4     5

Q4) How would you now rate your confidence in discussing scan requests with a radiologist?

*(1 = Not at all confident, 5 = Extremely confident)*

1       2     3     4     5

Q5) How would you now rate your ability to pick out key information from a patient’s notes?

*(1 = Not at all able, 5 = Extremely able)*

1       2     3     4     5

Q6) How would you now rate your ability to determine what additional information you may need about a patient prior to discussing a scan?  *(1 = Not at all able, 5 = Extremely able)*

1       2     3     4     5

Q7) How would you rate your baseline ability to synthesise a scan request in a logical manner?

*(1 = Not at all able, 5 = Extremely able)*

1       2     3   4     5

Q8) How would you now rate your ability to deal with a scan request being declined by a radiologist?  *(1 = Not at all able, 5 = Extremely able)*

1       2     3     4     5

Q9) Did you gain what you wanted to/ fulfill your objectives for the session?   Yes              No

Q10) How much would you recommend the session to your peers?

*(1= Definitely would not recommend, 5 = Would definitely recommend)*

1       2     3     4     **5**

How would you rate the session materials (E.g. cases)?

*(1 = Not very useful, 5 = Extremely useful)*

1       2     3   4     5            NA

What was good about this session?

What could be improved?
